# Risk Factors for Amoxicillin-Clavulanate Resistance in Community-Onset Urinary Tract Infections Caused by *Escherichia coli* or *Klebsiella pneumoniae*: The Role of Prior Exposure to Fluoroquinolones

**DOI:** 10.3390/antibiotics10050582

**Published:** 2021-05-14

**Authors:** Javier Martínez-Casanova, Silvia Gómez-Zorrilla, Nuria Prim, Agustina Dal Molin, Daniel Echeverría-Esnal, María Pilar Gracia-Arnillas, Elena Sendra, Robert Güerri-Fernández, Xavier Durán-Jordà, Eduardo Padilla, Juan Pablo Horcajada, Santiago Grau, on behalf of the PROA-PSMAR Group

**Affiliations:** 1 Infectious Pathology and Antimicrobials Research Group (IPAR), Pharmacy Department, Institut Hospital del Mar d’Investigacions Mèdiques (IMIM), Hospital del Mar, 08003 Barcelona, Spain; javier.marticasa@gmail.com (J.M.-C.); dechevarria@parcdesalutmar.cat (D.E.-E.); sgrau@hospitaldelmar.cat (S.G.); 2 Infectious Pathology and Antimicrobials Research Group (IPAR), Infectious Diseases Department, Institut Hospital del Mar d’Investigacions Mèdiques (IMIM), Universitat Autònoma de Barcelona (UAB), CEXS—Universitat Pompeu Fabra, Hospital del Mar, 08003 Barcelona, Spain; mdal@psmar.cat (A.D.M.); esendraalvarez@parcdesalutmar.cat (E.S.); rguerri@parcdesalutmar.cat (R.G.-F.); jhorcajada@hospitaldelmar.cat (J.P.H.); 3 Microbiology Service, Infectious Pathology and Antimicrobials Research Group (IPAR), Laboratoi de Referència de Catalunya, Institut Hospital del Mar d’Investigacions Mèdiques (IMIM), Universitat Autònoma de Barcelona (UAB), CEXS—Universitat Pompeu Fabra, Hospital del Mar, 08003 Barcelona, Spain; nuriaprim@gmail.com (N.P.); epadillal@lrc.cat (E.P.); 4 Critical Care Department, GREPAC, Institut Hospital del Mar d’Investigacions Mèdiques (IMIM), Hospital del Mar, 08003 Barcelona, Spain; mgraciaa@psmar.cat; 5 Scientific, Statistics and Technical Department, Institut Hospital del Mar d’Investigacions Mèdiques (IMIM), Hospital del Mar, 08003 Barcelona, Spain; xduran@imim.es

**Keywords:** amoxicillin/clavulanate, urinary tract infection (UTI), *Enterobacterales*, antimicrobial resistance, fluoroquinolones, emergency department

## Abstract

Background: High rates of amoxicillin-clavulanate (AMC) resistance among Enterobacterales isolated from urinary tract infections (UTIs) were observed in our area. The aim of this study was to identify risk factors associated with AMC resistance in patients with community-onset UTI in emergency departments (EDs). Methods: A retrospective study was performed of all ED patients with positive urine cultures for *Escherichia coli* or *Klebsiella pneumoniae* in a Spanish tertiary-care hospital. Results: 330 urine cultures in all were included: 261 (79.1%) for *E. coli* and 69 (20.90%) for *K. pneumonia.* Rates of AMC resistance were 14.94% and 34.78%, respectively. UTI was clinically confirmed in 212 (64.24%) cases. Previous antimicrobial exposure was independently associated with AMC resistance development in *E. coli* and *K. pneumoniae* urinary isolates (OR = 2.94, 95% CI = 1.55–5.58). Analyses of infected patients revealed that previous exposure to fluoroquinolones (OR = 3.33, 95% CI = 1.10–10.12, *p* = 0.034) and to AMC (OR = 5.68, 95% CI = 1.97–16.44, *p* = 0.001) was significantly associated with isolation of AMC-resistant strains. Conclusions: Prior antibiotic exposure, particularly to AMC or fluoroquinolones, was the only independent risk factor associated with development of AMC resistance in *E. coli* and *K. pneumoniae* urinary isolates from patients attending the ED.

## 1. Introduction

Urinary tract infection (UTI) is a common reason for attending hospital emergency departments (ED). In the United States, UTIs represented more than 3 million visits to ED [1], making it one of the most common reasons for prescribing empirical antibiotics [2,3]. In Spain, medical emergency visits due to infectious diseases represent more than 14% of all attended emergencies. UTIs are the second most frequent infection with 2517 visits/year (22.1%), surpassed only by respiratory tract infections with 3678 (32.3%) [2]. *Escherichia coli (E. coli*) is the most frequent (>60%) uropathogen responsible for this clinical condition, followed by *Klebsiella pneumoniae (K. pneumoniae)* (10%) [4,5].

There is general concern about increasing antibiotic resistance among uropathogens, partly related to the extensive use of antimicrobials [5]. This has reduced the empirical therapeutic options in many settings [6]. In our area, current guidelines no longer recommend quinolones or trimethoprim/sulfamethoxazole for empirical use [4]. Amoxicillin/clavulanate (AMC) is the most prescribed antimicrobial (in 26.5% of the cases) to treat community-onset UTIs (CO-UTI) [2]; in Spain, a striking increase in resistance of almost 15% has been observed in UTI isolates in just 5 years [7]. Taking into account that; following the EUCAST guidelines, the MIC established to define Enterobacterales as sensitive or resistant to AMC depends on whether it is a complicated or uncomplicated UTI; this data should be analyzed with caution before recommending no to use AMC in this setting [8].

*E. coli* and *K. pneumoniae* are of particular concern due to their high capacity to acquire extended-spectrum β-lactamases (ESBLs) that confer bacterial resistance to most β-lactam antibiotics [6,9]. ESBLs production often show cross-resistance to other group of antibiotics, being particularly worrisome the close relationship between ESBL production and fluoroquinolone resistance [10]. Conversely, the association between the use of fluoroquinolones and the development of ESBLs has been less evident.

Our institution periodically updates its antimicrobial guidelines, based on local microbiological data, within the framework of the Antimicrobial Stewardship Program. We noticed an upwards trend in AMC resistance in *Enterobacterales* urinary isolates, which could jeopardize its empirical use. Since AMC was one of the recommended empirical antimicrobial agents at our hospital for treatment of CA-UTI, we made it a priority to review the appropriateness of its empirical use in the ED setting. The aim of the present work was to investigate risk factors associated with AMC resistance in community-onset, ED-diagnosed UTIs caused by *E. coli* and *K. pneumoniae*.

## 2. Results

### 2.1. Epidemiological Data

A total of 330 urine cultures positive for *E. coli* and *K. pneumoniae* were collected during the study period. The median age of patients was 75.0 (47–85) years, and most were female (237 (71.82%)). Almost two-thirds of patients had at least one comorbidity (201 (60.91%)); of these, 56.72% (114/201) had more than two comorbidities. The most prevalent being diabetes and renal failure, present in 85 (25.76%) and 76 (23.03%) patients, respectively.

Asymptomatic bacteriuria was identified in 118 (35.76%) cases, and clinically confirmed UTI in 212 (64.24%) cases. Regarding the clinical presentation of UTI, 74 (34.91%) were cystitis and 138 (65.09%) complicated UTIs, of which 76 (55.07%) were pyelonephritis, 50 (36.23%) prostatitis, and the remaining cases were 7 orchiepididymitis and 5 urinary device-related infections.

Most of the cases were community-acquired (229 (69.39%)). Fifty-nine (17.88%) patients had a history of recurrent UTI. Fifty-four (16.36%) patients carried an indwelling urinary device, of which 31 (57.41%) were urinary catheters. Overall, 49 (14.85%) patients had been admitted to hospital and 110 (33.33%) had received a course of antibiotics in the 3 months prior to ED admission.

### 2.2. Microbiological Data

The most frequently isolated uropathogen was *E. coli* (261 (79.10%)), followed by *K. pneumoniae* (69 (20.90%)). Overall AMC resistance was 17.88% (59/330). The MIC distributions for AMC were: ≤8 mg/L in 271 (82.12%) isolates, >8 mg/L and ≤32 mg/L in 34 (10.30%), and >32 mg/L in 25 (7.58%). AMC resistance rates in *E. coli* and *K. pneumoniae* isolates were 14.94% (39/261) and 28.96% (20/69), respectively. Fluoroquinolone resistance rates of *E. coli* and *K. pneumoniae* isolates were 28.35% (74/261) and 34.78% (24/69), respectively. Overall, there were 12.12% (40/330) ESBL carriers, corresponding to 9.96% (26/261) of all *E. coli* isolates and 20.29% (14/69) of *K. pneumoniae*. Half of the ESBL carriers were AMC-resistant (52.50% (21/40)). Ten AMC-resistant isolates were ESBL *E. coli*; three of these had also an AmpC β-lactamase hyperproduction profile. Two enterobacterial isolates had an acquired AmpC β-lactamase (1 *E. coli* and 1 *K. pneumoniae*). Two *K. pneumoniae* isolates had a carbapenemase (New Delhi metallo-β-lactamase and OXA-48).

### 2.3. Risk Factors for AMC Resistance in All Patients

The baseline characteristics of included patients are described in Table 1. Patients with AMC-resistant isolates were older than those with susceptible isolates (median age, 80 (67–85) years vs. 72 (42–85) years, *p* = 0.004)) and significantly more likely to have higher Charlson index scores (median points, 5 (3–7) vs. 2 (0–5); *p* < 0.001), malignant disease (*p* < 0.001), chronic kidney disease (*p* = 0.002), and neurological disease (*p* = 0.023). AMC-resistant isolates were also significantly associated with prior antibiotic exposure (57.63% vs. 28.04%; *p* < 0.001), urinary device use (20.34% vs. 7.01%; *p* = 0.005), prior hospital stay (23.73% vs. 12.92%; *p* = 0.043), and immunosuppression (18.64% vs. 8.49%; *p* = 0.031). 

Table 2 shows multivariate logistic regression analyses of variables associated with AMC resistance. Previous exposure to AMC or fluoroquinolones was the only variable associated with AMC resistance in *E. coli* and *K. pneumoniae* urinary isolates (OR = 2.94, 95% CI = 1.55–5.58, *p* = 0.001).

### 2.4. Risk Factors for AMC Resistance in Infected Patients

Factors related to AMC resistance were analyzed in the ‘confirmed UTI’ patient subset, and shown in Table 3. Age, Charlson index, SAPS II score, diabetes, COPD, malignant disease, immunosuppression, indwelling urinary catheter use, previous hospital stay, previous use of AMC, carbapenems or any other antibiotic, complicated UTI, and CO-HCA were significantly associated with AMC resistance.

Multivariate analysis showed that the two variables independently associated with AMC resistance in this subgroup of patients were: previous exposure to fluoroquinolones (OR = 3.33, 95% CI = 1.10–10.12, *p* = 0.034) and previous exposure to AMC (OR = 5.68, 95% CI = 1.97–16.44, *p* = 0.001) (Table 4).

## 3. Discussion

Increasing antibiotic resistance among uropathogens [5] can lead to inappropriate empirical treatment of UTI. In this challenging scenario, guidelines based on local antimicrobial susceptibility profiles are essential to select appropriate empirical antibiotic treatments. Given the high rates of AMC resistance detected in *Enterobacterales* (EUCAST criteria) while updating our local UTI guidelines, we conducted a retrospective study to assess risk factors for AMC resistance in urinary enterobacterial isolates from adult patients attending the ED. As expected, the isolates were mainly *E. coli* and *K. pneumoniae*.

AMC nonsusceptibility was detected in 14.94% of *E. coli* and in 28.96% of *K. pneumoniae*. A multicentric study performed in Spain showed that AMC-resistance in *E. coli* was due to OXA-1 β-lactamase production in one fourth of the isolates [11], followed by hyperproduction of penicillinases, hyperproduction of the chromosomic AmpC β-lactamase, production of acquired AmpC, and production of inhibitor-resistant TEM. Currently, carbapenemases may also contribute, especially OXA-48 type in our environment [9]. Thus, AMC resistance may be acquired by several mechanisms that interplay in a complex epidemiological background, including clonal spread, dissemination of different bla genes, and mutations in individual isolates as a response to selective antimicrobial pressure [11].

Risk factors for antibiotic resistance in UTI isolates in community settings have been evaluated previously [10,12,13]. None of these reports focused specifically on risk factors for AMC resistance. In our report, previous antibiotic exposure was the only independent risk factor associated with AMC resistance in both *E. coli* and *K. pneumoniae* isolates. When analyzed only the subgroup of patients with symptomatic UTI, previous use of either AMC or fluoroquinolones were significantly associated.

It has been widely reported that antimicrobial use exerts selective pressure that leads to an increase in antimicrobial resistance [10,13,14]. It is reasonable to suppose that individual exposure to AMC results in selection of AMC-resistant bacteria. V. Leflon-Guibot et al. specifically studied this association in a recent study in hospitalized patients with confirmed UTI [15]. They conclude that exposure to AMC was a risk factor for selection of AMC-resistant *E. coli* isolates. They did not however evaluate previous use of other antibiotics [15].

The association between fluoroquinolones and the development of resistance in β lactams may not be as straightforward as in the case of AMC. Fluoroquinolones have been associated with the clonal expansion of different multidrug-resistant (MDR) bacteria, ranging from methicillin-resistant *Staphylococcus aureus* to ESBL-carrying *Enterobacterales* such as the *E. coli* ST131-H30 subclone [10,12,16,17]. Double-serine fluoroquinolone resistance mutations in the DNA gyrase and topoisomerase IV genes are present in the major successful clones [16,17]. These specific mutations seem to impact fitness favorably, providing clones with increased spreading capacity in fluoroquinolone environments. Other mutations in minor clones do not seem to contribute to increased fitness [16,17]. Fluoroquinolone resistance has also been involved in the spread of other enterobacterial clones, regardless of their resistance profile to other antibiotics [17,18]. Hence, the association between fluoroquinolone resistance and AMC observed in our study may be partly driven by clonal spread, despite the low number of ESBL carriers and other MDR isolates. The high rates of fluoroquinolone resistance in our isolates may support the previously reported role of these broad-spectrum antibiotics in bacterial evolutionary success [16,17,19].

Mechanisms other than chromosomal mutations may also have contributed. Plasmid-mediated quinolone-resistance can carry other antimicrobial resistance genes such as ESBLs [16,17,20], while multidrug chromosomal efflux pumps have the ability to actively remove different families of antibacterial drugs, including fluoroquinolones [14,17,21,22]. Indeed, previous fluoroquinolone use has been associated with the emergence of multidrug efflux pumps [14,23], which cause cross-resistance between sublethal concentrations of fluoroquinolones and other antibiotic families, including AMC [24].

Moreover, β-lactamase production and porin decrease are well-recognized mechanisms of resistance against β-lactam antibiotics among Gram-negative bacteria. However, we did not perform molecular studies to analyze the presence of any of these fluoroquinolone resistance determinants. 

According to the EUCAST guidelines, the MIC breakpoints to categorize AMC susceptibility in Enterobacterales causing UTIs are either ≤8 mg/L or ≤32 mg/L, depending on whether the infection leads to a complicated or an uncomplicated UTI, respectively [8]. However, microbiologists usually do not have enough clinical information in this respect and the episode is often considered a complicated UTI. In the present study, the AMC MIC value was above 8 mg/L in 17.88% of enterobacterial isolates and were consequently all categorized as resistant. However, 10.30% (34/330) had MICs of either 16 or 32 mg/L, and could have been categorized as susceptible or resistant to AMC depending on whether the physician considered the UTI episode as complicated or uncomplicated. AMC doses of 250 mg/125 mg have been related to maximum urine concentrations between 647 and 1547 mg/L and 150 and 439 mg/L for amoxicillin and clavulanate, respectively [25]. These values would explain why current AMC doses of 1 g/125 mg could be sufficient for the treatment of most UTIs caused by these strains. Unfortunately, these “in-between” MIC values may overestimate AMC resistance and lead many clinicians to refrain from using this antibiotic in certain clinical situations where it could be used safely. Replacing AMC with broader spectrum antibiotics would have a negative impact on the ecological background and therefore on stewardship programs. Clinical uncertainty is further increased by the absence of consensus on definitions of complicated UTI in the IDSA and ESCMID guidelines. The medical literature uses many different definitions of complicated UTI [26]. Such heterogeneity makes it even more difficult to apply the EUCAST criteria, although this committee has recently included definitions of these processes [27]. In this context, it is essential to reach international consensus on the standard definitions for establishing complicated and uncomplicated UTI. Furthermore, we consider that having two different AMC breakpoints for enterobacterial isolates in UTI can be misleading in some clinical settings. This EUCAST criterion would probably benefit from revision.

The present study has some limitations. First, it was a retrospective data analysis, and dependent on the accuracy of the clinical histories collected. Second, we did not conduct a clonal study, which would have been useful to confirm a possible association between AMC resistance and certain clones. Molecular studies would also have been useful to study resistance determinants. Third, it was a single-centre study, conducted in the ED setting and focused only on *E. coli* and *K. pneumoniae* isolates; our results may not be generalizable to other geographical areas, healthcare settings or infections caused by other bacteria. Fourth, prior amoxicillin/clavulanate use in the subgroup of infected patients, variable that is independently associated with AMC resistance, show a rather large 95% CI. Therefore, the association should be verified in new studies with a larger sample size. Finally, it was conducted over a short period (2 months) with no follow-up of the clinical consequences of the observed resistance. Large-sample, prospective multicenter studies are needed to better understand the extent and outcomes of infections caused by AMC-resistant Gram-negative bacteria.

## 4. Materials and Methods

### 4.1. Study Design

This was a retrospective observational cohort study performed over a two-month period (November-December 2017) at the Hospital del Mar, a 420-bed tertiary-care university teaching hospital in Barcelona (Spain). Using computer-generated microbiological data, all adult patients (>18-years-old) attending the ED during this period with positive urine cultures for *E. coli* or *K. pneumoniae* were included. Cases were defined as patients with AMC-resistant isolates; controls were those with AMC-susceptible isolates. This paper was written following the STROBE guidelines for observational studies [28].

### 4.2. Data Collection and Definitions

Demographic, clinical, and epidemiological data were collected by examining the hospital and primary care medical and nursing records. Comorbidities considered were diabetes mellitus, chronic obstructive pulmonary disease (COPD), cardiovascular disease, chronic kidney disease, chronic liver disease, neurological disorders, malignant disease, human immunodeficiency virus infection, and immunosuppression. Patients were considered to have malignant disease when malignancy was diagnosed within the previous 5 years or were receiving specific oncological therapy. Patients were considered immunosuppressed when they received chemotherapy, radiotherapy, systemic corticosteroids at a dose higher than 10 mg of prednisone per day or equivalent, or other immunosuppressive agents in the 3 months prior to ED admission. The Charlson comorbidity index [29] and the simplified acute physiologic score (SAPSII) [30] were recorded upon ED arrival. Variables related to the patient’s urinary history, such as urinary tract abnormalities, recurrent UTI, or urinary device use in the preceding month were also recorded. Patients with at least 3 UTI episodes in the previous 12 months were considered recurrent UTI. Regarding site of acquisition, community-onset healthcare-associated (CO-HCA) episodes were defined as those fulfilling any of the Friedman criteria [31], and otherwise as community-acquired. Prior antibiotic exposure was defined as administration of antibiotics for more than 48 h in the previous 3 months, and prior hospital stay as previous hospitalization in the last 3 months before ED admission.

Definition of infection was established according to the Centers for Disease Control and Prevention criteria [32]. All episodes were reviewed retrospectively by two authors (J.M.-C. and A.D.M.) and classified as confirmed UTI or asymptomatic bacteriuria. Controversies were double-checked by a third investigator (S.G.-Z.). Among confirmed UTIs, cystitis was considered to have uncomplicated UTI; other infections (prostatitis, pyelonephritis, and orchiepididymitis) were considered as complicated UTI.

### 4.3. Microbiological Data

Urine cultures were performed as part of clinical routine following standard laboratory procedures. Cultures with growth yielding ≥10^5^ colony-forming units/mL of a single bacterial type in a urine sample collected midstream were considered positive. Pyuria was defined as ≥10 white blood cells/mm^3^. Only one urine culture per patient was included.

Antibiotic susceptibility testing was performed by microdilution using MicroScan panels (Beckman Coulter, Brea, CA, USA) in an automated WalkAway system (Beckman Coulter). Results were interpreted following European Committee for Antimicrobial Susceptibility Testing (EUCAST) 2019 guidelines [8]. Isolates with AMC minimum inhibitory concentration (MIC) values >8 mg/L were considered resistant. Phenotypic detection of ESBLs and carbapenemases was in accordance with EUCAST recommendations [33]. Molecular confirmation of carbapenemases was by real time PCR, using LightMix^®^ modular carbapenemase kits (TIB Molbiol, Berlin, Germany) in a LightCycler 480 II instrument (Roche Diagnostics, Rotkreuz, Switzerland).

### 4.4. Statistical Analysis

Continuous variables were expressed as median and interquartile range (IQR); categorical variables were expressed as counts and percentages. Continuous variables were compared using the Mann–Whitney U-test and categorical variables by the Fisher’s exact test.

An analysis of all patients was made for AMC resistance, and a separate one for the ‘confirmed UTI’ subgroup. Univariate analysis was performed first. Variables showing statistically significant differences between susceptible and resistant microorganisms were included in the multivariate model. Clinically relevant variables considered by the literature to be potential confounders were also studied in the multivariate model, even if they did not show significant differences in the previous step. Multivariate analysis was carried out using a logistic regression model to estimate the risk factors involved in the development of antibiotic resistance. Strength of association was expressed as odds ratios (ORs). A two-sided *p*-value <0.05 was statistically significant. All analyses were performed using STATA v. 15.1.

## 5. Conclusions

Prior to antibiotic exposure, particularly to AMC and fluoroquinolones, within the previous three months, was independently associated with development of AMC-resistant *E. coli* and *K. pneumoniae* strains in urinary isolates of patients attending the ED. These results could be used to assess the appropriateness of AMC as empirical treatment in similar UTI cases attending the emergency department A general reduction in the use of AMC and fluoroquinolones should be encouraged to reduce antimicrobial resistance.

## Figures and Tables

**Table 1:** Baseline characteristics of 330 patients according to amoxicillin/clavulanate susceptibility in urinary isolates.

Baseline Characteristics	Susceptible Isolates *n* = 271	Resistant Isolates *n* = 59	*p*-Value
	*n* (%)	*n* (%)	
Age (years), median (IQR)	72 (42–85)	80 (67–85)	0.004
Sex male	69 (25.46)	24 (40.68)	0.025
Charlson Comorbidity index at admission, median (IQR)	2 (0–5)	5 (3–7)	<0.001
SAPS II at admission, median (IQR)	28 (20–34)	33 (28–41)	<0.001
Main underlying diseases			
Diabetes	64 (23.62)	21 (35.59)	0.070
Chronic pulmonary disease	18 (6.64)	8 (13.56)	0.104
Cardiovascular disease	38 (14.02)	11 (18.64)	0.418
Renal failure	53 (19.56)	23 (38.98)	0.002
Chronic liver disease	16 (5.90)	4 (6.78)	0.766
Neurological disease	42 (15.50)	17 (28.81)	0.023
Malignant disease	18 (6.64)	14 (23.73)	<0.001
Human immunodeficiency virus	4 (1.48)	2 (3.39)	0.292
Immunosuppression	23 (8.49)	11 (18.64)	0.031
Recurrent UTI history	43 (15.87)	16 (27.12)	0.059
Indwelling urinary catheter	19 (7.01)	12 (20.34)	0.005
Other indwelling urinary devices	17 (6.27)	6 (10.17)	0.269
Prior hospital stay (3 months)	35 (12.92)	14 (23.73)	0.043
Prior antibiotic use (3 months)			
Amoxicillin/clavulanate	21 (7.75)	14 (23.73)	0.002
Piperacillin/tazobactam	4 (1.48)	0 (0.00)	1.000
Cephalosporin	12 (4.43)	6 (10.17)	0.107
Carbapenem	4 (1.48)	4 (6.78)	0.037
Fluoroquinolone	28 (10.33)	17 (28.81)	0.001
Fosfomycin	19 (7.01)	5 (8.47)	0.781
Linezolid	3 (1.11)	1 (1.69)	0.547
Aminoglycoside	0 (0.00)	1 (1.69)	0.179
Aztreonam	3 (1.11)	0 (0.00)	1.000
Other antibiotic	21 (7.75)	7 (11.86)	0.306
Any antibiotic	76 (28.04)	34 (57.63)	<0.001
Clinical features			
Asymptomatic bacteriuria	98 (36.16)	20 (33.90)	0.767
Clinical infection	173 (63.84)	39 (66.10)	0.767
Current bacteremic UTI Pitt Score, median (IQR)	13 (4.80) 0 (0–1)	5 (8.48) 0.5 (0–0)	0.337 0.411
Type of acquisition			
CA-UTI	200 (73.80)	29 (49.15)	<0.001
CO-HCA UTI	71 (26.20)	30 (50.85)	<0.001

Data are presented as absolute number (%), unless otherwise specified. Continuous variables were compared using the Mann–Whitney U-test and categorical variables by using the Fisher’s exact test. Abbreviations: IQR: interquartile range; SAPS II: simplified acute physiology score II; UTI: urinary tract infection; CA-UTI: community acquired urinary tract infection; CO-HCA UTI: community onset-healthcare associated urinary tract infection.

**Table 2:** Multivariate logistic regression analysis of parameters predicting amoxicillin/clavulanate resistance in all patients.

Amoxicillin/Clavulanate Resistance in All Patients with *E. coli* or *K. pneumoniae* Urinary Isolate (*n* = 330; Amoxicillin/Clavulanate Resistance Episodes = 59)		
Parameter	Adjusted OR (95% CI)	*p*-Value
Age	1.01 (0.99–1.03)	0.269
Charlson Comorbidity Index	1.04 (0.93–1.17)	0.457
SAPS II	1.02 (0.97–1.06)	0.496
Immunosuppression	2.05 (0.85–4.93)	0.110
Indwelling urinary catheter	1.84 (0.75–4.51)	0.183
Prior hospital stay (3 months)	0.54 (0.21–1.39)	0.168
Prior fluoroquinolones or amoxicillin/clavulanate use (3 months)	2.94 (1.55–5.58)	0.001
CO-HCA UTI	1.75 (0.78–3.92)	0.177

Multivariate logistic regression model was used for examining independent variables associated with amoxicillin/clavulanate resistance, using stepwise automatic variable selection procedure. All patients of the study (*n* = 330) were included. Abbreviations: 95% CI: 95% confidence interval, SAPS II: simplified acute physiology score II; CO-HCA UTI: community onset-healthcare associated urinary tract infection.

**Table 3:** Baseline characteristics of 212 infected patients according to amoxicillin/clavulanate-susceptibility in urinary isolates.

Baseline Characteristics	Susceptible Isolates *n* = 173	Resistant Isolates *n* = 39	*p*-Value
	*n* (%)	*n* (%)	
Age (years), median (IQR)	56 (37–80)	76 (61–83)	0.002
Sex male	52(30.06)	18 (46.15)	0.061
Charlson Comorbidity Index at admission, median (IQR)	1 (0–3)	5 (2–7)	<0.001
SAPS II at admission, median (IQR)	24 (19–30)	31 (26–39)	<0.001
Main underlying diseases			
Diabetes	31 (17.92)	13 (33.33)	0.032
Chronic pulmonary disease	4 (2.31)	4 (10.26)	0.040
Cardiovascular disease	14 (8.09)	7 (17.95)	0.076
Renal failure	28 (16.19)	10 (25.64)	0.171
Chronic liver disease	8 (4.62)	4 (10.26)	0.240
Neurological disease	20 (11.56)	9 (23.08)	0.072
Malignant disease	9 (5.20)	11 (28.21)	<0.001
Human immunodeficiency virus	3 (1.73)	1 (2.56)	0.559
Immunosuppression	16 (9.25)	9 (23.08)	0.025
Recurrent UTI history	32 (18.50)	11 (28.21)	0.189
Indwelling urinary catheter	12 (6.94)	9 (23.08)	0.005
Other indwelling urinary devices	14 (8.09)	3 (7.69)	1.000
Prior hospital stay (3 months)	20 (11.56)	12 (30.77)	0.005
Prior antibiotic use (3 months)			
Amoxicillin/clavulanate	14 (8.09)	12 (30.77)	<0.001
Piperacillin/tazobactam	3 (1.73)	0 (0.00)	1.000
Cephalosporin	10 (5.78)	6 (15.39)	0.084
Carbapenem	2 (1.16)	4 (10.26)	0.011
Fluoroquinolone	18 (10.41)	9 (23.08)	0.058
Fosfomycin	15 (8.67)	4 (10.26)	0.758
Linezolid	1 (0.58)	1 (2.56)	0.335
Aminoglycoside	0 (0.00)	1 (2.56)	0.184
Aztreonam	2 (1.16)	0 (0.00)	1.000
Other antibiotic	12 (6.94)	4 (10.26)	0.503
Any antibiotic	51 (29.48)	23 (58.97)	0.001
Clinical features			
Complicated UTI	106 (61.27)	32 (82.05)	0.016
Current bacteremic UTI Pitt Score, median (IQR)	11 (6.36) 0 (0–1)	5 (12.82) 0.5 (0–1)	0.182 0.373
Type of acquisition			
CA-UTI	130 (75.15)	17 (43.59)	<0.001
CO-HCA UTI	43 (24.86)	22 (56.41)	<0.001

Data are presented as absolute number (%), unless otherwise specified. Continuous variables were compared using the Mann–Whitney U-test and categorical variables by using the Fisher’s exact test. Abbreviations: IQR: interquartile range; SAPS II: simplified acute physiology score II; UTI: urinary tract infection; CA-UTI: community acquired urinary tract infection; CO-HCA UTI: community onset-healthcare associated urinary tract infection.

**Table 4:** Multivariate logistic regression analysis of parameters predicting amoxicillin/clavulanate resistance in infected patients. Amoxicillin/clavulanate resistance in infected patients with *E. coli* or *K. pneumoniae* urinary isolate (*n* = 212; amoxicillin/clavulanate resistance episodes = 39).

Parameter	Adjusted OR (95% CI)	*p*-Value
Age	1.01 (0.98–1.04)	0.705
SAPS II	1.03 (0.96–1.10)	0.414
Chronic pulmonary disease	3.35 (0.65–17.34)	0.150
Renal failure	0.38 (0.11–1.27)	0.116
Neurological disease	3.08 (0.95–9.96)	0.061
Malignant disease	2.12 (0.56–8.11)	0.272
Immunosuppression	3.24 (0.89–11.87)	0.076
Indwelling urinary catheter	1.13 (0.32–3.98)	0.850
Prior hospital stay (3 months)	0.84 (0.23–3.09)	0.792
Prior fluoroquinolone use (3 months)	3.33 (1.10–10.12)	0.034
Prior amoxicillin/clavulanate use (3 months)	5.68 (1.97–16.44)	0.001
Prior carbapenem use (3 months)	2.26 (0.22–23.31)	0.493
CO-HCA UTI	1.77 (0.57–6.48)	0.320

Multivariate logistic regression model was used for examining independent variables associated with amoxicillin/clavulanate resistance, using stepwise automatic variable selection procedure. Only patients with clinical infection (UTI), (*n* = 212) were included. Abbreviations: 95% CI: 95% confidence interval; SAPS II: simplified acute physiology score II; CO-HCA UTI: community onset-healthcare associated urinary tract infection; UTI: urinary tract infection.

## Data Availability

The data presented in this study are available on request from the corresponding author.

## References

[1] Long B., Koyfman A. (2018). The Emergency Department Diagnosis and Management of Urinary Tract Infection. Emerg. Med. Clin..

[2] De Zarate M.M.O., Del Castillo J.G., Jiménez A.J., Salmerón P.P., Roca F.L., Tey J.G., Borras M.R.C., Grinspan M.R., Lamberechts E.J.G., Esparza C.I. (2013). Estudio INFURG-SEMES: Epidemiología de las infecciones atendidas en los servicios de urgencias hospitalarios y evolución durante la última década. Emergencias.

[3] May L., Mullins P., Pines J. (2014). Demographic and treatment patterns for infections in ambulatory settings in the United states, 2006–2010. Acad. Emerg. Med..

[4] Gupta K., Hooton T.M., Naber K.G., Wullt B., Colgan R., Miller L.G., Moran G.J., Nicolle L.E., Raz R., Schaeffer A.J. (2011). International Clinical Practice Guidelines for the Treatment of Acute Uncomplicated Cystitis and Pyelonephritis in Women: A 2010n Update by the Infectious Diseases Society of America and the European Society for Microbiology and Infectious Diseases. J. Bacteriol. Res..

[5] Zhanel G.G., Hisanaga T.L., Laing N.M., DeCorby M.R., Nichol K.A., Palatnick L.P., Johnson J., Noreddin A., Harding G.K., Nicolle L.E. (2005). Antibiotic resistance in outpatient urinary isolates: Final results from the North American Urinary Tract Infection Collaborative Alliance (NAUTICA). Int. J. Antimicrob. Agents.

[6] Meier S., Weber R., Zbinden R., Ruef C., Hasse B. (2011). Extended-spectrum β-lactamase-producing Gram-negative pathogens in community-acquired urinary tract infections: An increasing challenge for antimicrobial therapy. Infection.

[7] Bosch-Nicolau P., Falcó V., Viñado B., Andreu A., Len O., Almirante B., Pigrau C. (2017). A cohort study of risk factors that influence empirical treatment of patients with acute pyelonephritis. Antimicrob. Agents Chemother..

[8] The European Committee on Antimicrobial Susceptibility Testing (2019). Breakpoint Tables for Interpretation of MICs and Zone Diameters. Version 9.0. http://www.eucast.org.

[9] Pitout J.D.D., Peirano G., Kock M.M., Strydom K.A., Matsumura Y. (2020). The global ascendency of OXA-48-type carbapenemases. Clin. Microbiol. Rev..

[10] Colodner R., Rock W., Chazan B., Keller N., Guy N., Sakran W., Raz R. (2004). Risk factors for the development of extended-spectrum beta-lactamase- producing bacteria in nonhospitalized patients. Eur. J. Clin. Microbiol. Infect. Dis..

[11] Ortega A., Oteo J., Aranzamendi-Zaldumbide M., Bartolomé R.M., Bou G., Cercenado E., Conejo M.C., González-López J.J., Marín M., Martínez-Martínez L. (2012). Spanish multicenter study of the epidemiology and mechanisms of amoxicillin-clavulanate resistance in Escherichia coli. Antimicrob. Agents Chemother..

[12] Bischoff S., Walter T., Gerigk M., Ebert M., Vogelmann R. (2018). Empiric antibiotic therapy in urinary tract infection in patients with risk factors for antibiotic resistance in a German emergency department. BMC Infect. Dis..

[13] Howard A.J. (2001). Factors associated with antibiotic resistance in coliform organisms from community urinary tract infection in Wales. J. Antimicrob. Chemother..

[14] Nikaido H., Pagès J.M. (2012). Broad-specificity efflux pumps and their role in multidrug resistance of Gram-negative bacteria. FEMS Microbiol. Rev..

[15] Leflon-Guibout V. (2002). Exposure to co-amoxiclav as a risk factor for co-amoxiclav-resistant Escherichia coli urinary tract infection. J. Antimicrob. Chemother..

[16] Fuzi M., Rodriguez Baño J., Toth A. (2020). Global Evolution of Pathogenic Bacteria With Extensive Use of Fluoroquinolone Agents. Front. Microbiol..

[17] Redgrave L.S., Sutton S.B., Webber M.A., Piddock L.J.V. (2014). Fluoroquinolone resistance: Mechanisms, impact on bacteria, and role in evolutionary success. Trends Microbiol..

[18] Platell J.L., Trott D.J., Johnson J.R., Heisig P., Heisig A., Clabots C.R., Johnston B., Cobbold R.N. (2012). Prominence of an O75 clonal group (clonal complex 14) among non-st131 fluoroquinolone-resistant Escherichia coli causing extraintestinal infections in humans and dogs in Australia. Antimicrob. Agents Chemother..

[19] Marcusson L.L., Frimodt-Møller N., Hughes D. (2009). Interplay in the selection of fluoroquinolone resistance and bacterial fitness. PLoS Pathog..

[20] Fuzi M. (2016). Dissimilar fitness associated with resistance to fluoroquinolones influences clonal dynamics of various multiresistant bacteria. Front. Microbiol..

[21] Poole K. (2007). Efflux pumps as antimicrobial resistance mechanisms. Ann. Med..

[22] Piddock L.J.V. (2006). Clinically relevant chromosomally encoded multidrug resistance efflux pumps in bacteria. Clin. Microbiol..

[23] Hasdemir U.O., Chevalier J., Nordmann P., Pagès J.M. (2004). Detection and prevalence of active drug efflux mechanism in various multidrug-resistant Klebsiella pneumoniae strains from Turkey. J. Clin. Microbiol..

[24] Adamus-Białek W., Wawszczak M., Arabski M., Majchrzak M., Gulba M., Jarych D., Parniewski P., Głuszek S. (2019). Ciprofloxacin, amoxicillin, and aminoglycosides stimulate genetic and phenotypic changes in uropathogenic Escherichia coli strains. Virulence.

[25] Weber D.J., Tolkoff-Rubin N.E., Rubin R.H. (1984). Amoxicillin and Potassium Clavulanate: An Antibiotic Combination Mechanism of Action, Pharmacokinetics, Antimicrobial Spectrum, Clinical Efficacy and Adverse Effects. J. Hum. Pharmacol. Drug Ther..

[26] Johansen T.E.B., Botto H., Cek M., Grabe M., Tenke P., Wagenlehner F.M.E., Naber K.G. (2011). Critical review of current definitions of urinary tract infections and proposal of an EAU/ESIU classification system. Int. J. Antimicrob. Agents.

[27] The European Committee on Antimicrobial Susceptibility Testing (2020). Breakpoint Tables for Interpretation of MICs and Zone Diameters. Version 10.0. http://www.eucast.org.

[28] Von Elm E., Altman D.G., Egger M., Pocock S.J., Gøtzsche P.C., Vandenbrouckef J.P. (2007). The Strengthening the Reporting of Observational Studies in Epidemiology (STROBE) Statement: Guidelines for reporting observational studies. Bull. World Health Organ..

[29] Charlson M.E., Pompei P., Ales K.L., MacKenzie C.R. (1987). A new method of classifying prognostic comorbidity in longitudinal studies: Development and validation. J. Chronic Dis..

[30] Le Gall J.R., Lemeshow S., Saulnier F. (1993). Simplified Acute Physiology Score (SAPS II) Based on a European/North American Multicenter Study. Jama.

[31] Friedman N.D. (2002). Health Care–Associated Bloodstream Infections in Adults: A Reason To Change the Accepted Definition of Community-Acquired Infections. Ann. Intern. Med..

[32] National Healthcare Safety Network (2020). CDC/NHSN Surveillance Definitions for Specific Types of Infections.

[33] The European Committee on Antimicrobial Susceptibility Testing (2017). EUCAST Guidelines for Detection of Resistance Mechanisms and Specific Resistances of Clinical and/or Epidemiological Importance. Version 2.0. http://www.eucast.org.

